# Multivariate box spline wavelets in higher-dimensional Sobolev spaces

**DOI:** 10.1186/s13660-018-1839-z

**Published:** 2018-09-19

**Authors:** Raj Kumar, Manish Chauhan

**Affiliations:** 1 Department of Mathematics, Kirori Mal College, University of Delhi, New Delhi, India; 2 Department of Mathematics, University of Delhi, New Delhi, India

**Keywords:** Wavelets, Box Splines, Multiresolution analysis, Sobolev space

## Abstract

We construct wavelets and derive a density condition of MRA in a higher-dimensional Sobolev space. We give necessary and sufficient conditions for orthonormality of wavelets in $H^{s}(\mathbb {R}^{d})$. We construct nonseparable orthonormal wavelets in a higher-dimensional Sobolev space by using multivariate box spline.

## Introduction

Box splines are refinable functions, and we can easily choose various directions to have a box spline function with a desired order of smoothness. Naturally, they have been used to construct various wavelet functions. Mathematically box splines offer an elegant toolbox for constructing a class of multidimensional elements with flexible shape and support. In multivariate setting, box splines are often considered as a generalization of B-splines [1]. Theoretically, the computational complexity of a box spline is lower than that of an equivalent B-spline, since its support is more compact and its total polynomial degree is lower. To investigate this potential in practice, several attempts were made. Recurrence relation [1, 2] is the most commonly used technique for evaluating box splines at an arbitrary position. There are many papers on multivariate spline wavelet theory, in particular, on orthogonal spline wavelets [3], compactly spline prewavelets [4–6], bivariate and trivariate compactly supported biorthogonal box spline wavelets [7, 8], and multivariate compactly supported tight wavelet frames [9].

Wavelets in a Sobolev space and their properties were instigated by Bastin et al. [10, 11], Dayong and Dengfeng [12], and Pathak [13]. Regular compactly supported wavelets and compactly supported wavelets of integer order in a Sobolev space by B-spline are given in [10, 11]. Further, bivariate box splines in a Sobolev space were introduced in [14].

Inspired by the works mentioned, in this paper, we study nonseparable wavelets in a higher-dimensional Sobolev space by using a multivariate box spline. To the best of our knowledge, no previous studies of multivariate box spline wavelets exist in higher-dimensional Sobolev spaces. This paper is organized as follows. In Sect. 2, we hereby present construction of wavelets and density conditions of MRA in a higher-dimensional Sobolev space. Also, we give necessary and sufficient conditions for the orthonormality of wavelets in $H^{s}(\mathbb {R}^{d})$. In Sect. 3, we construct nonseparable wavelets in a higher-dimensional Sobolev space by using a multivariate box spline.

### Sobolev space $H^{s}(\mathbb{R}^{d})$

For any real number *s*, the Sobolev space $H^{s}(\mathbb {R}^{d})$ consists of tempered distributions in $S'(\mathbb {R}^{d})$ such that

$$\Vert f \Vert _{s}^{2}:=\frac{1}{(2\pi)^{d}} \int_{\mathbb {R}^{d}}\bigl(1+ \Vert \xi \Vert ^{2} \bigr)^{s} \bigl\vert \hat{f}(\xi) \bigr\vert ^{2}\,d\xi, $$

where $\|\cdot\|$ denotes the Euclidean norm in $\mathbb {R}^{d}$, and the corresponding inner product is

$$\langle f, g \rangle_{s}:=\frac{1}{(2\pi)^{d}} \int_{\mathbb {R}^{d}}\bigl(1+ \Vert \xi \Vert ^{2} \bigr)^{s}\hat{f}(\xi)\overline{\hat{g}(\xi)}\,d\xi. $$

The Fourier transform *f̂* of $f\in L^{1}(\mathbb {R}^{d})$ is defined as

$$\hat{f}(\xi):= \int_{\mathbb {R}^{d}}e^{-i \langle x,\xi \rangle}f(x)\,dx, $$

where $\langle x,\xi \rangle$ is the inner product of two vectors *x* and *ξ* in $\mathbb {R}^{d}$.

## Multiresolution analysis

To adapt classical theory of MRA over $H^{s}(\mathbb {R}^{d})$, we first derive an orthonormality and density condition. The main problem is that $H^{s}$-norm is not dilation invariant. We also don’t achieve orhtonormality at each level of dilation by a single scaling function. This lead us to a more general construction of MRA, where the scaling function depends on the level of dilation. Throughout this paper, the superscript *j* of a function $\varphi^{(j)}$ represents level *j*.

### Proposition 2.1

*If* *s* *is a real number*, $\varphi^{(j)}\in H^{s}(\mathbb {R}^{d}) $, *and* *j* *is an integer*, *then the distributions* $\varphi _{j,k}^{(j)}(x)=2^{{jd}/2}\varphi^{(j)}(2^{j}x-k), k\in\mathbb {Z}^{d}$, *are orthonormal in* $H^{s}(\mathbb {R}^{d}) $ *iff*

1 $$\begin{aligned} \sum_{k\in\mathbb {Z}^{d}} \bigl(1+2^{2j} \Vert \xi+2k\pi \Vert ^{2} \bigr)^{s} \bigl\vert \hat{\varphi}^{(j)}(\xi+2k\pi) \bigr\vert ^{2}=1 \end{aligned}$$

*almost everywhere*. *It follows that we have the bound*

$$\bigl\vert \hat{\varphi}^{(j)} \bigl(2^{-j}\xi \bigr) \bigr\vert \leq \bigl(1+ \Vert \xi \Vert ^{2} \bigr)^{-s/2}. $$

### Proof

Since $\varphi_{j,k}^{(j)}(t)\in H^{s}(\mathbb {R}^{d})$, the series

$$M(\xi)=\sum_{r\in\mathbb {Z}^{d}}{ \bigl\vert \hat{\varphi}^{(j)}(\xi+2\pi r) \bigr\vert }^{2}{ \bigl(1+2^{2j}{ \bigl\Vert (\xi+2\pi r) \bigr\Vert }^{2} \bigr)}^{s} $$

converges almost everywhere, belongs to $\mathbb {L}_{\mathrm{ loc}}^{1}(\mathbb {R}^{d})$, and is $2\pi\mathbb {Z}^{d}$-periodic, that is, $M(\xi )\in L^{1}(\mathbb {T}^{d})$, where $\mathbb {T}^{d}=[0,2\pi]^{d}$ is the *d*-dimensional torus. Moreover, for every $l\in\mathbb {Z}^{d}$, we have

$$\begin{aligned} &\int_{\mathbb {T}^{d}}M(\xi)e^{-i\langle\xi,(k-l)\rangle}\,d\xi \\ &\quad=\sum_{r\in\mathbb {Z}^{d}} \int_{\mathbb {T}^{d}}{ \bigl\vert \hat{\varphi}^{(j)}(\xi +2\pi r) \bigr\vert }^{2}{\bigl(1+2^{2j}{ \bigl\Vert (\xi+2\pi r) \bigr\Vert }^{2}\bigr)}^{s}e^{-i\langle\xi ,(k-l)\rangle}\,d\xi \\ &\quad= \int_{\mathbb {R}^{d}}{ \bigl\vert \hat{\varphi}^{(j)}(\nu) \bigr\vert }^{2}{\bigl(1+2^{2j}{ \bigl\Vert (\nu) \bigr\Vert }^{2}\bigr)}^{s}e^{-i\langle\nu,(k-l)\rangle}\,d\nu \\ &\quad=2^{-jd} \int_{\mathbb {R}^{d}}{ \bigl\vert \hat{\varphi}^{(j)} \bigl(2^{-j}u\bigr) \bigr\vert }^{2}{\bigl(1+{ \Vert u \Vert }^{2}\bigr)}^{s}e^{-i 2^{-j}\langle u,(k-l)\rangle}\,du \\ &\quad= \int_{\mathbb {R}^{d}}{\bigl(1+{ \Vert u \Vert }^{2} \bigr)}^{s}e^{-i 2^{-j}\langle u,k\rangle }2^{-jd/2}\hat{\varphi}^{(j)} \bigl(2^{-j}u\bigr) \overline{e^{-i 2^{-j}\langle u,l\rangle}2^{-jd/2}\hat{\varphi}^{(j)}\bigl(2^{-j}u\bigr)}\,du \\ &\quad= \int_{\mathbb {R}^{d}}{\bigl(1+{ \Vert u \Vert }^{2} \bigr)}^{s}\mathcal{F} \bigl[ \varphi _{j,k}^{(j)}(t) \bigr](u)\mathcal{F} \bigl[ \varphi_{j,l}^{(j)}(t) \bigr](u)\,du \\ &\quad={(2\pi)^{d}}\bigl\langle \varphi_{j,k}^{(j)}(t), \varphi _{j,l}^{(j)}(t)\bigr\rangle _{s}. \end{aligned}$$

Since $\{1/{(2\pi)^{d}}e^{-i\langle\xi,(k-l)\rangle}:k,l\in \mathbb {Z}^{d}\}$ is an orthonormal basis for $L^{2}(\mathbb {T}^{d})$, we have

$$\begin{aligned} \frac{1}{{(2\pi)}^{d}} \int_{\mathbb {T}^{d}}M(\xi)e^{-i\langle\xi ,(k-l)\rangle}\,d\xi=\bigl\langle \varphi_{j,k}^{(j)}(t), \varphi _{j,l}^{(j)}(t) \bigr\rangle _{s}=\delta_{k,l} \end{aligned}$$

if $M(\xi)=1$.

From (1) we get

$$\bigl(1+2^{2j} \Vert \xi \Vert ^{2} \bigr)^{s} \bigl\vert \hat{\varphi}^{(j)}(\xi) \bigr\vert ^{2}\leq1, $$

which implies

$$\bigl\vert \hat{\varphi}^{(j)}(\xi) \bigr\vert \leq \bigl(1+2^{2j} \Vert \xi \Vert ^{2} \bigr)^{-s/2}. $$

 □

### Proposition 2.2

*Let* $\varphi^{(j)}, j\in\mathbb {Z}$, *be a sequence of elements of* $H^{s}(\mathbb {R}^{d})$ *such that*, *for every* *j*, *the distributions* $\varphi _{j,k}^{(j)}(x)=2^{{jd}/2}\varphi^{(j)}(2^{j}x-k), k\in\mathbb {Z}^{d}$, *are orthonormal in* $H^{s}(\mathbb {R}^{d})$. *If* $P_{j}$ *is the orthogonal projection from* $H^{s}(\mathbb {R}^{d})$ *onto* $V_{j}:=\overline{\operatorname{span}}\{ \varphi_{j,k}^{(j)}: k\in\mathbb {Z}^{d}\}$, *then*, *for every* $h\in H^{s}(\mathbb {R}^{d})$, *we have*

$$\lim_{j\rightarrow{+\infty}} \biggl( \Vert P_{j}h \Vert _{s}^{2}-\frac{1}{(2\pi)^{d}} \int _{\mathbb {R}^{d}}\bigl(1+ \Vert \xi \Vert ^{2} \bigr)^{2s} \bigl\vert \hat{h}(\xi) \bigr\vert ^{2} \bigl\vert \hat{\varphi}^{(j)}\bigl(2^{-j}\xi\bigr) \bigr\vert ^{2}\,d\xi \biggr)=0. $$

*Moreover*, *if there are* $A,\alpha>0$ *such that*

$$\int_{\mathbb {R}^{d}}\bigl(1+ \Vert \xi \Vert \bigr)^{\alpha} \bigl\vert \hat{\varphi}^{(j)}(\xi) \bigr\vert ^{2}\,d\xi \leq A $$

*for every* $j\leq0$, *then* $\bigcap_{j={-\infty}}^{j=\infty}V_{j}=\{0\}^{d}$.

### Proof

Let us prove the first part with $h\in C_{0}^{\infty}(\mathbb {R}^{d})$. By the definition of $P_{j}$ we get

$$\Vert P_{j}h \Vert _{s}^{2}=\sum _{k\in{\mathbb {Z}^{d}}} \bigl\vert {\bigl\langle h, \varphi _{j,k}^{(j)}\bigr\rangle }_{s} \bigr\vert ^{2}=\frac{2^{-jd}}{(2\pi)^{2d}}\sum_{k\in\mathbb {Z}^{d}} \biggl\vert \int_{\mathbb {R}^{d}}\bigl(1+ \Vert \xi \Vert ^{2} \bigr)^{s}\hat{h}(\xi)\overline{\hat{\varphi}^{(j)} \bigl(2^{-j}\xi\bigr)}e^{i2^{-j}\langle k,\xi\rangle}\,d\xi \biggr\vert ^{2}. $$

Moreover, since *h* and $\varphi^{(j)}$ belong to $H^{s}(\mathbb {R}^{d})$,

$$\begin{aligned} & \int_{\mathbb {R}^{d}}\bigl(1+ \Vert \xi \Vert ^{2} \bigr)^{s}\hat{h}(\xi)\overline{\hat{\varphi}^{(j)} \bigl(2^{-j}\xi\bigr)}e^{i2^{-j}\langle k,\xi\rangle}\,d\xi \\ &\quad = \int_{]0,{2^{j}}2\pi[^{d}}e^{i2^{-j}\langle k,\xi\rangle}\sum_{p\in \mathbb {Z}^{d}} \bigl(1+ \bigl\Vert \xi+2^{j}2\pi p \bigr\Vert ^{2} \bigr)^{s}\hat{h}\bigl(\xi+2^{j}2\pi p\bigr)\overline{\hat{\varphi}^{(j)}\bigl(2^{-j}\xi+2\pi p\bigr)}\,d\xi. \end{aligned}$$

Hence, using the Parseval formula in $L^{2}({ ]0,2^{j}2\pi [}^{d})$, we get

$$\begin{aligned} & \Vert P_{j}h \Vert _{s}^{2} \\ & \quad=\frac{1}{(2\pi)^{d}} \int_{]0,{2^{j}}2\pi[^{d}} \biggl\vert \sum_{p\in\mathbb {Z}^{d}} \bigl(1+ \bigl\Vert \xi+2^{j}2\pi p \bigr\Vert ^{2} \bigr)^{s}\hat{h}\bigl(\xi+2^{j}2\pi p\bigr)\overline{\hat{\varphi}^{(j)}\bigl(2^{-j}\xi+2\pi p\bigr)} \biggr\vert ^{2}\,d\xi \\ &\quad =\frac{1}{(2\pi)^{d}}\sum_{q\in\mathbb {Z}^{d}} \int_{\mathbb {R}^{d}}\bigl(1+ \Vert \xi \Vert ^{2} \bigr)^{s}\bigl(1+ \bigl\Vert \xi+2^{j}2\pi q \bigr\Vert ^{2}\bigr)^{s}\hat{h}(\xi)\overline{\hat{\varphi}^{(j)}\bigl(2^{-j}\xi\bigr)} \\ &\qquad{} \times\overline{\hat{h}\bigl(\xi+2^{j}2\pi q\bigr)}\hat{\varphi}^{(j)}\bigl(2^{-j}\xi +2\pi q\bigr) \,d\xi \\ & \quad=\frac{1}{(2\pi)^{d}} \int_{\mathbb {R}^{d}}\bigl(1+ \Vert \xi \Vert ^{2} \bigr)^{2s} \bigl\vert \hat{h}(\xi ) \bigr\vert ^{2} \bigl\vert \hat{\varphi}^{(j)}\bigl(2^{-j}\xi\bigr) \bigr\vert ^{2} \\ &\qquad{} +\frac{1}{(2\pi)^{d}}\sum_{q\in\mathbb {Z}^{d}\backslash\{0\}^{d}} \int _{\mathbb {R}^{d}}\bigl(1+ \Vert \xi \Vert ^{2} \bigr)^{s}\bigl(1+ \bigl\Vert \xi+2^{j}2\pi q \bigr\Vert ^{2}\bigr)^{s}\hat{h}(\xi )\overline{\hat{\varphi}^{(j)} \bigl(2^{-j}\xi\bigr)} \\ & \qquad{}\times\overline{\hat{h}\bigl(\xi+2^{j}2\pi q\bigr)}\hat{\varphi}^{(j)}\bigl(2^{-j}\xi +2\pi q\bigr) \,d\xi. \end{aligned}$$

The term associated with $q=\{0\}^{d}, \{0\}^{d}=(0,0,\ldots,0)\in\mathbb {Z}^{d} $ is used as an approximation for $\|P_{j}h\|_{s}^{2}$. Using Proposition 2.1, the inequality $|\varphi^{(j)}(2^{-j}\xi)|\leq(1+\|\xi\|^{2})^{-s/2}$, and the fact that *ĥ* belongs to the Schwartz space $S(\mathbb {R}^{d})$ (i.e., $|\hat{h}(\xi)|\leq C(1+\|\xi\|^{2})^{-\alpha}$ for any $\alpha>0$), we obtain that the sum of the other ones is bounded by

$$\begin{aligned} &\sum_{q\in\mathbb {Z}^{d}\backslash\{0\}^{d}} \int_{\mathbb {R}^{d} }\bigl(1+ \Vert \xi \Vert ^{2} \bigr)^{s/2}\bigl(1+ \bigl\Vert \xi+2^{j}2\pi q \bigr\Vert ^{2}\bigr)^{s/2} \bigl\vert \hat{h}(\xi)\overline{\hat{h} \bigl(\xi +2^{j}2\pi q\bigr)} \bigr\vert \,d\xi \\ &\quad\leq C\sum_{q\in\mathbb {Z}^{d}\backslash\{0\}^{d}}\frac{1}{(1+ \Vert 2^{j}2\pi q \Vert ^{2})^{2}} \int_{\mathbb {R}^{d} }\frac{1}{(1+ \Vert \xi \Vert ^{2})^{2}}\,d\xi \\ &\quad\leq C\sum_{q\in\mathbb {Z}^{d}\backslash\{0\}^{d}}\frac{1}{( \Vert 2^{j}2\pi q \Vert ^{2})^{2}} \int_{\mathbb {R}^{d} }\frac{1}{(1+ \Vert \xi \Vert ^{2})^{2}}\,d\xi \\ &\quad\leq C2^{-4(j+1)} \biggl(\sum_{q\in\mathbb {Z}^{d}\backslash\{0\}^{d}} \frac {1}{ \vert q \vert ^{2}} \biggr) \int_{\mathbb {R}^{d} }\frac{1}{(1+ \Vert \xi \Vert ^{2})^{2}}\,d\xi, \end{aligned}$$

where $|q|= (\sum_{r=1}^{d}|q_{r}|^{2} )^{1/2}, q=(q_{1},q_{2},\ldots ,q_{d})\in\mathbb {Z}^{d}$. This expression converges to 0 as $j\rightarrow{+\infty}$.

Now let $h\in H^{s}(\mathbb {R}^{d})$. Recall the inequality

$$\Vert f+g \Vert ^{2}\leq(1+\varepsilon) \Vert f \Vert ^{2}+ \biggl(1+\frac{1}{\varepsilon} \biggr) \Vert g \Vert ^{2}, $$

which is valid for every $\varepsilon>0$ and any seminorm. For any $\chi \in C_{0}^{\infty}(\mathbb {R}^{d})$, we have

$$\begin{aligned} &\Vert P_{j}h \Vert _{s}^{2}-\frac{1}{(2\pi)^{d}} \int_{\mathbb {R}^{d}}\bigl(1+ \Vert \xi \Vert ^{2} \bigr)^{2s} \bigl\vert \hat{h}(\xi) \bigr\vert ^{2} \bigl\vert \hat{\varphi}^{(j)}\bigl(2^{-j}\xi\bigr) \bigr\vert ^{2}\,d\xi \\ &\quad\leq(1+\varepsilon) \Vert P_{j}\chi \Vert _{s}^{2}+ \biggl(1+\frac{1}{\varepsilon} \biggr) \bigl\Vert P_{j}(h-\chi) \bigr\Vert ^{2} \\ &\qquad{} -\frac{1}{(2\pi)^{d}(1+\varepsilon)} \int_{\mathbb {R}^{d}}\bigl(1+ \Vert \xi \Vert ^{2} \bigr)^{2s} \bigl\vert \hat{\chi}(\xi) \bigr\vert ^{2} \bigl\vert \hat{\varphi}^{(j)}\bigl(2^{-j}\xi\bigr) \bigr\vert ^{2}\,d\xi \\ &\qquad{} +\frac{1}{(2\pi)^{d}(\varepsilon)} \int_{\mathbb {R}^{d}}\bigl(1+ \Vert \xi \Vert ^{2} \bigr)^{2s} \bigl\vert \hat{h}(\xi)-\hat{\chi}(\xi) \bigr\vert ^{2} \bigl\vert \hat{\varphi}^{(j)}\bigl(2^{-j}\xi \bigr) \bigr\vert ^{2}\,d\xi \\ &\quad\leq(1+\varepsilon) \biggl( \Vert P_{j}\chi \Vert _{s}^{2}-\frac{1}{(2\pi)^{d}} \int _{\mathbb {R}^{d}}\bigl(1+ \Vert \xi \Vert ^{2} \bigr)^{2s} \bigl\vert \hat{\chi}(\xi) \bigr\vert ^{2} \bigl\vert \hat{\varphi}^{(j)}\bigl(2^{-j}\xi\bigr) \bigr\vert ^{2}\,d\xi \biggr) \\ &\qquad{} + \biggl(1+\frac{2}{\varepsilon} \biggr) \Vert h-\chi \Vert _{s}^{2}+ \biggl(1+\varepsilon-\frac{1}{(1+\varepsilon)} \biggr) \Vert \chi \Vert _{s}^{2}. \end{aligned}$$

By the same way, we can obtain a similar lower bound. To prove that the left-hand side converges to 0 as *j* converges to +∞, we first take *ε* sufficiently small. Then we choose *χ* approximating *h* and finally *j* large.

For the second part, we have to prove that, for every $h\in C_{0}^{\infty}(\mathbb {R}^{d})$, $P_{j}h$ converges to zero in $H^{s}(\mathbb {R}^{d})$ as $j\rightarrow{-\infty}$. We use the last expression of $\|P_{j}h\|_{s}$ obtained previously. We first estimate the sum over *q* without the integral. By the Cauchy–Schwarz inequality and Proposition 2.1 we have

$$\begin{aligned} & \sum_{q\in\mathbb {Z}^{d}}\bigl(1+\bigl\Vert \xi+2^{j}2\pi q \bigr\Vert ^{2}\bigr)^{s} \bigl\vert \hat{h}\bigl(\xi+2^{j}2\pi q\bigr)\overline{\hat{\varphi}^{(j)}\bigl(2^{-j}\xi+2\pi q\bigr)} \bigr\vert \\ &\quad\leq \biggl(\sum_{q\in\mathbb {Z}^{d}}\bigl(1+ \bigl\Vert \xi+2^{j}2\pi q \bigr\Vert ^{2}\bigr)^{s} \bigl\vert \hat{h}\bigl(\xi +2^{j}2\pi q\bigr) \bigr\vert ^{2} \biggr)^{1/2}. \end{aligned}$$

We know that

$$\begin{aligned} \sum_{q\in\mathbb {Z}^{d}}\bigl(1+ \bigl\Vert \xi+2^{j}2\pi q \bigr\Vert ^{2}\bigr)^{s} \bigl\vert \hat{h}\bigl(\xi+2^{j}2\pi q\bigr) \bigr\vert ^{2} \bigl(2^{j+1}\pi\bigr)^{d}\rightarrow \int_{\mathbb {R}^{d}}\bigl(1+ \Vert \xi \Vert ^{2} \bigr)^{s} \bigl\vert \hat{h}(\xi) \bigr\vert ^{2}\,d\xi \end{aligned}$$

if $j\leq-1$. It follows that

$$\begin{aligned} \Vert P_{j}h \Vert _{s}^{2}\leq{}& \frac{1}{(2\pi)^{d}} \int_{\mathbb {R}^{d}}\bigl(1+ \Vert \xi \Vert ^{2} \bigr)^{s} \bigl\vert \hat{h}(\xi)\hat{\varphi}^{(j)} \bigl(2^{-j}\xi\bigr) \bigr\vert 2^{-jd}C \Vert h \Vert _{s}\,d\xi \\ \leq&{}\frac{2^{-jd}C \Vert h \Vert _{s}}{(2\pi)^{d}} \biggl( \int_{\mathbb {R}^{d}}\bigl(1+2^{-j} \Vert \xi \Vert \bigr)^{\alpha} \bigl\vert \hat{\varphi}^{(j)}\bigl(2^{-j} \xi\bigr) \bigr\vert ^{2}\,d\xi \biggr)^{1/2} \\ &{}\times \biggl( \int_{\mathbb {R}^{d}}\bigl(1+2^{-j} \Vert \xi \Vert \bigr)^{-\alpha}\bigl(1+ \Vert \xi \Vert ^{2} \bigr)^{2s} \bigl\vert \hat{h}(\xi) \bigr\vert ^{2}\,d\xi \biggr)^{1/2} \\ \leq{}&\frac{C\sqrt{A} \Vert h \Vert _{s}}{(2\pi)^{d}} \biggl( \int_{\mathbb {R}^{d}}\bigl(1+2^{-j} \Vert \xi \Vert \bigr)^{-\alpha}\bigl(1+ \Vert \xi \Vert ^{2} \bigr)^{2s} \bigl\vert \hat{h}(\xi) \bigr\vert ^{2}\,d\xi \biggr)^{1/2}. \end{aligned}$$

The last expression converges to zero as *j* converges to −∞. □

Now we construct wavelets in $H^{s}(\mathbb {R}^{d})$ with the help of previous propositions.

By definition, $V_{j}$ is the set of all $f\in H^{s}(\mathbb {R}^{d})$ such that

$$\hat{f}(\xi)=m \bigl(2^{-j}\xi \bigr)\hat{\varphi}^{(j)} \bigl(2^{-j}\xi \bigr), $$

where $m\in L_{\mathrm{loc}}^{2} (\mathbb {R}^{d})$ is $2\pi\mathbb {Z}^{d}$-periodic. This follows immediately from the fact that the Fourier transform of $2^{{jd}/2}\varphi ^{(j)}(2^{j}x-k)$ is

$$2^{{-jd}/2}e^{-i2^{-j}\langle k,\xi\rangle}\hat{\varphi}^{(j)} \bigl(2^{-j}\xi\bigr). $$

We have $V_{j}\subset V_{j+1}$ for every $j\in\mathbb {Z}^{d}$ iff there are $2\pi\mathbb {Z}^{d}$-periodic functions $m_{0}^{(j)}\in L_{\mathrm{loc}}^{2} (\mathbb {R}^{d})$ such that the following scale relation holds:

2 $$\begin{aligned} \hat{\varphi}^{(j)}(2\xi)=m_{0}^{(j+1)} ( \xi )\hat{\varphi}^{(j+1)} (\xi ); \end{aligned}$$

moreover, $\varphi^{(j)}$ and $\varphi^{(j+1)}$ satisfy the hypothesis of Proposition 2.1. Now, using our theorems and propositions, we develop the definition of MRA in $H^{s}(\mathbb {R}^{d})$.

### Definition 2.3

Let *s* be a real number. The MRA of $H^{s}(\mathbb {R}^{d})$ is a sequence $V_{j}, j\in\mathbb {Z}$, of closed linear subspaces of $H^{s}(\mathbb {R}^{d})$ such that

- $V_{j}\subset V_{j+1}$,
- $\bigcup_{j=-\infty}^{j=\infty}V_{j}= H^{s}(\mathbb {R}^{d})$,
- $\bigcap_{j=-\infty}^{j=\infty}V_{j}= \{0\}^{d}$, and
- for every *j*, there is a function $\varphi^{(j)}$ such that the distributions $2^{{jd}/2}\varphi^{(j)}(2^{j}x-k)$, $k\in\mathbb {Z}^{d}$, form an orthonormal basis for $V_{j}$.

Before giving a necessary condition for the orthonormality, we define $E_{d}:=\{0,1\}^{d}$ as the unit cube in the *d*-dimensional Euclidean space.

### Theorem 2.4

*If* $\varphi^{(j)}$ *and* $\varphi^{(j+1)}$ *satisfy the hypothesis of Proposition *2.1, *then*

$$\sum_{q=0}^{2^{d}-1} \bigl\vert m_{0}^{(j+1)}(\xi+\gamma_{q}\pi) \bigr\vert =1,\quad \gamma_{q}\in E_{d}, q=1,2,\ldots, 2^{d}-1. $$

### Proof

We know from Proposition 2.1 that if the system is orthonormal, then

$$\begin{aligned} \delta_{k,l}={}&\bigl\langle \varphi_{j,k}^{(j)}, \varphi_{j,l}^{(j)} \bigr\rangle _{s} \\ ={}&\frac{2^{-jd}}{(2\pi)^{d}} \int_{\mathbb {R}^{d}}{ \bigl\vert \hat{\varphi}^{(j)} \bigl(2^{-j}\xi\bigr) \bigr\vert }^{2}e^{-i2^{-j} \langle\xi,(k-l) \rangle}{ \bigl(1+{ \Vert \xi \Vert }^{2}\bigr)}^{s}\,d\xi \\ ={}&\frac{1}{(2\pi)^{d}} \int_{\mathbb {R}^{d}}{ \bigl\vert \hat{\varphi}^{(j)}(u) \bigr\vert }^{2}e^{-i \langle u,(k-l) \rangle}{\bigl(1+2^{2j}{ \Vert u \Vert }^{2}\bigr)}^{s}\,du \\ ={}&\frac{1}{(2\pi)^{d}} \int_{\mathbb {R}^{d}}{ \bigl\vert m_{0}^{(j+1)}(u/2) \bigr\vert }^{2}{ \bigl\vert \hat{\varphi}^{(j+1)}(u/2) \bigr\vert }^{2}e^{-i \langle u,(k-l) \rangle}{\bigl(1+2^{2j}{ \Vert u \Vert }^{2}\bigr)}^{s}\,du \\ ={}&\frac{2^{d}}{(2\pi)^{d}} \int_{\mathbb {R}^{d}}{ \bigl\vert m_{0}^{(j+1)}(\nu) \bigr\vert }^{2}{ \bigl\vert \hat{\varphi}^{(j+1)}(\nu) \bigr\vert }^{2}e^{-i 2 \langle\nu,(k-l) \rangle}{\bigl(1+2^{2(j+1)}{ \Vert \nu \Vert }^{2}\bigr)}^{s}d\nu \\ ={}&\frac{1}{(\pi)^{d}} \int_{\mathbb {T}^{d}}{ \bigl\vert m_{0}^{(j+1)}(\nu) \bigr\vert }^{2}\sum_{r\in\mathbb {Z}^{d}}{ \bigl\vert \hat{\varphi}^{(j+1)}(\nu+2\pi r) \bigr\vert }^{2} \\ &{} \times{\bigl(1+2^{2(j+1)}{ \Vert {\nu+2\pi r} \Vert }^{2} \bigr)}^{s}e^{-i 2 \langle\nu,(k-l) \rangle}\,d\nu \\ ={}&\frac{1}{(\pi)^{d}} \int_{\mathbb {T}^{d}}{ \bigl\vert m_{0}^{(j+1)}(\nu) \bigr\vert }^{2}e^{-i 2 \langle\nu,(k-l) \rangle}\,d\nu \\ ={}&\frac{1}{(\pi)^{d}} \int_{[0,\pi)^{d}}\sum_{q=0}^{2^{d}-1} \bigl\vert m_{0}^{(j+1)}(\nu +\gamma_{q}\pi) \bigr\vert ^{2}e^{-i 2 \langle\nu,(k-l) \rangle}\,d\nu, \end{aligned}$$

which implies that

$$\sum_{q=0}^{2^{d}-1} \bigl\vert m_{0}^{(j+1)}(\xi+\gamma_{q}\pi) \bigr\vert ^{2}=1,\quad \gamma_{q}\in E_{d}, $$

if $k=l$. □

With the help of (2) and Theorem 2.4, we may define $\varphi^{(j)}$ by

3 $$\begin{aligned} \hat{\varphi}^{(j)}(\xi)&= m_{0}^{(j+1)}(\xi/2)\hat{\varphi}^{(j+1)}(\xi /2) \\ &=\prod_{t=1}^{J} m_{0}^{(j+t)} \bigl(\xi/2^{t}\bigr)\hat{\varphi}^{(j+J)}\bigl(\xi/2^{J} \bigr) \\ &=\cdots=\frac{1}{(1+ \Vert \xi \Vert ^{2})^{s/2}}\prod_{t=1}^{+\infty} m_{0}^{(j+t)}\bigl(\xi/2^{t}\bigr) \end{aligned}$$

for $j\in\mathbb {Z}$. For $V_{j}$, let $W_{j}$ be the orthogonal complement of $V_{j}$ in $V_{j+1}$. We have

4 $$\begin{aligned} \psi^{(j)}_{j,k,p}:=2^{jd/2} \psi^{(j)}_{p}\bigl(2^{j}x-k\bigr) \in V_{j+1} \end{aligned}$$

if there are $2\pi\mathbb {Z}^{d}$-periodic functions $m^{(j)}_{1},m^{(j)}_{2},\ldots,m^{(j)}_{2^{d}-1}\in L^{2}_{\mathrm{loc}}(\mathbb {R}^{d})$ such that

$$\hat{\psi}^{(j)}_{p}\bigl(2^{-j}\xi \bigr)=m_{p}^{(j+1)}\bigl(2^{-j-1}\xi\bigr)\hat{\varphi}^{(j+1)}\bigl(2^{-j-1}\xi\bigr),\quad p=1,2,\ldots, 2^{d}-1. $$

### Theorem 2.5

*The distributions* $\psi_{j,k,p}^{(j)}(x)=2^{jd/2}\psi_{p}^{(j)}(2^{j}x-k)$ *are orthonormal if*

$$\sum_{q=0}^{2^{d}-1} \bigl\vert m_{p}^{(j+1 )}(\xi+\gamma_{q}\pi) \bigr\vert =1,\quad \gamma_{q}\in E_{d}, \forall p=1,2,\ldots, 2^{d}-1, $$

*and they are orthogonal to* $V_{j}$ *if*

5 $$ \sum_{q=0}^{2^{d}-1}m_{p}^{(j+1)}( \xi+\gamma_{q}\pi)\overline{m_{0}^{(j+1)}(\xi + \gamma_{q}\pi)}=0,\quad \gamma_{q}\in E_{d}, \forall p=1,2,\ldots, 2^{d}-1. $$

### Proof

$$\begin{aligned} &\bigl\langle \psi_{j,k,p}^{(j)},\psi_{j,l,p}^{(j)} \bigr\rangle _{s} \\ &\quad=\frac{2^{-jd}}{(2\pi)^{d}} \int_{\mathbb {R}^{d}}{ \bigl\vert \hat{\psi}_{p}^{(j)} \bigl(2^{-j}\xi\bigr) \bigr\vert }^{2}e^{-i2^{-j} \langle\xi,(k-l) \rangle }{ \bigl(1+{ \Vert \xi \Vert }^{2}\bigr)}^{s}\,d\xi \\ &\quad=\frac{1}{(2\pi)^{d}} \int_{\mathbb {R}^{d}}{ \bigl\vert \hat{\psi}_{p}^{(j)}(u) \bigr\vert }^{2}e^{-i \langle u,(k-l) \rangle}{\bigl(1+2^{2j}{ \Vert u \Vert }^{2}\bigr)}^{s}\,du \\ &\quad=\frac{1}{(2\pi)^{d}} \int_{\mathbb {R}^{d}}{ \bigl\vert m_{p}^{(j+1)}(u/2) \bigr\vert }^{2}{ \bigl\vert \hat{\varphi}^{(j+1)}(u/2) \bigr\vert }^{2}e^{-i \langle u,(k-l) \rangle }{\bigl(1+2^{2j}{ \Vert u \Vert }^{2}\bigr)}^{s}\,du \\ &\quad=\frac{2^{d}}{(2\pi)^{d}} \int_{\mathbb {R}^{d}}{ \bigl\vert m_{p}^{(j+1)}(\nu) \bigr\vert }^{2}{ \bigl\vert \hat{\varphi}^{(j+1)}(\nu) \bigr\vert }^{2}e^{-i2 \langle\nu,(k-l) \rangle}{\bigl(1+2^{2(j+1)}{ \Vert \nu \Vert }^{2}\bigr)}^{s}d\nu \\ &\quad=\frac{1}{(\pi)^{d}} \int_{\mathbb {T}^{d}}{ \bigl\vert m_{p}^{(j+1)}(\nu) \bigr\vert }^{2}\sum_{r\in \mathbb {Z}^{d}}{ \bigl\vert \hat{\varphi}^{(j+1)}(\nu+2\pi r) \bigr\vert }^{2} \\ &\qquad{} \times{\bigl(1+2^{2(j+1)}{ \Vert {\nu+2\pi r} \Vert }^{2} \bigr)}^{s}e^{-i 2 \langle\nu ,(k-l) \rangle}\,d\nu \\ &\quad=\frac{1}{(\pi)^{d}} \int_{\mathbb {T}^{d}}{ \bigl\vert m_{p}^{(j+1)}(\nu) \bigr\vert }^{2}e^{-i 2 \langle\nu,(k-l) \rangle}\,d\nu \\ &\quad=\frac{1}{(\pi)^{d}} \int_{[0,\pi)^{d}}\sum_{q=1}^{2^{d}-1} { \bigl\vert m_{p}^{(j+1)}(\nu+\gamma_{q}\pi) \bigr\vert }^{2}e^{-i 2 \langle\nu,(k-l) \rangle}\,d\nu \\ &\quad=\frac{1}{(\pi)^{d}} \int_{[0,\pi)^{d}}e^{-i2 \langle\nu,(k-l) \rangle}\,d\nu. \end{aligned}$$

Therefore

$$\begin{aligned} \bigl\langle \psi_{j,k,p}^{(j)},&\psi_{j,l,p}^{(j)} \bigr\rangle _{s}=1 \end{aligned}$$

if $\sum_{q=0}^{2^{d}-1}|m_{p}^{(j+1 )}(\xi+\gamma_{q}\pi)|=1, \gamma_{q}\in E_{d}$, and $k=l$.

Now we prove second part of the theorem:

$$\begin{aligned} 0={}&\bigl\langle \psi_{j,k,p}^{(j)},\varphi_{j,l,p}^{(j)} \bigr\rangle _{s} \\ ={}&\frac{2^{-jd}}{(2\pi)^{d}} \int_{\mathbb {R}^{d}}{\bigl(1+{ \Vert \xi \Vert }^{2} \bigr)}^{s}{\hat{\psi}_{p}^{(j)}\bigl(2^{-j} \xi\bigr)}\overline{\hat{\varphi}^{(j)}\bigl(2^{-j}\xi \bigr)}e^{-i2^{-j} \langle\xi,(k-l) \rangle}\,d\xi \\ ={}&\frac{1}{(2\pi)^{d}} \int_{\mathbb {R}^{d}}{\bigl(1+2^{2j}{ \Vert \xi \Vert }^{2}\bigr)}^{s}{ m_{p}^{(j+1)}(\xi/2)} \overline{m_{0}^{(j+1)}(\xi/2)} { \bigl\vert \hat{\varphi}^{(j+1)}(\xi/2) \bigr\vert ^{2}}e^{-i \langle\xi,(k-l) \rangle }\,d\xi \\ ={}&\frac{2^{d}}{(2\pi)^{d}} \int_{\mathbb {R}^{d}}{\bigl(1+2^{2(j+1)}{ \Vert \xi \Vert }^{2}\bigr)}^{s}{ m_{p}^{(j+1)}(\xi)} \overline{m_{0}^{(j+1)}(\xi)} { \bigl\vert \hat{\varphi}^{(j+1)}(\xi) \bigr\vert ^{2}}e^{-i2 \langle\xi,(k-l) \rangle}\,d\xi \\ ={}&\frac{1}{(\pi)^{d}} \int_{\mathbb {T}^{d}}m_{p}^{(j+1)}(\xi) \overline{m_{0}^{(j+1)}(\xi)} \\ &{} \times\sum_{r\in\mathbb {Z}^{d}}{\bigl(1+2^{2(j+1)}{ \Vert \xi+2r\pi \Vert }^{2}\bigr)}^{s}{ \bigl\vert \hat{\varphi}^{(j+1)}(\xi+2r\pi) \bigr\vert ^{2}}e^{-i2 \langle\xi,(k-l) \rangle }\,d \xi \\ ={}&\frac{1}{(\pi)^{d}} \int_{[0,\pi)^{d}}\sum_{q=1}^{2^{d}-1} m_{p}^{(j+1)}(\xi+\gamma_{q}\pi) \overline{m_{0}^{(j+1)}(\xi+\gamma_{q}\pi )}e^{-i 2 \langle\xi,(k-l) \rangle}\,d\xi, \end{aligned}$$

which implies

$$\sum_{q=0}^{2^{d}-1}m_{p}^{(j+1)}( \xi+\gamma_{q}\pi)\overline{m_{0}^{(j+1)}(\xi + \gamma_{q}\pi)}=0,\quad \gamma_{q}\in E_{d}, \forall p=1,2,\ldots, 2^{d}-1. $$

 □

Now we define unitary matrix with the help of our theorems,

6 $$ \begin{bmatrix} m_{0}^{(j)}(\xi+\gamma_{0}\pi) & m_{0}^{(j)}(\xi+\gamma_{1}\pi) & \cdots& m_{0}^{(j)}(\xi+\gamma_{2^{d}-1}\pi) \\ m_{1}^{(j)}(\xi+\gamma_{0}\pi) & m_{1}^{(j)}(\xi+\gamma_{1}\pi) & \cdots& m_{1}^{(j)}(\xi+\gamma_{2^{d}-1}\pi) \\ \ddots& \ddots& \ddots& \ddots&\\ m_{2^{d}-1}^{(j)}(\xi+\gamma_{0}\pi) & m_{2^{d}-1}^{(j)}(\xi+\gamma_{1}\pi) & \cdots& m_{2^{d}-1}^{(j)}(\xi+\gamma_{2^{d}-1}\pi) \end{bmatrix} . $$

### Theorem 2.6

*Suppose that the scaling function* $\varphi^{(j)}, j\in \mathbb {Z}$, *generate an MRA* $\{V_{j}\}$ *of* $H^{s}(\mathbb {R}^{d})$ *and* $\varphi _{j,k}^{(j)}, k\in\mathbb {Z}^{d}$, *form an orthonormal basis for* $V_{j}, j\in\mathbb {Z}$. *Suppose that*, *for each* $j\in\mathbb {Z}, m^{(j)}_{p}$ *for* $p=1,2,\ldots,2^{d}-1$ *are such that matrix* (6) *is unitary*. *Define* $\psi_{j,k,p}^{(j)}$ *by* (4) *for* $p=1,2,\ldots,2^{d}-1$ *and* $j\in\mathbb {Z}$. *Then* $W_{j}=W_{j,1}\oplus W_{j,2}\oplus\cdots\oplus W_{j,2^{d}-1}$ *with* $W_{j,p}=\overline{\operatorname{span}}\{2^{jd/2}\psi ^{(j)}_{p}(2^{j}x-k): k\in\mathbb {Z}\}, p=1,2,\ldots,2^{d}-1$, *is perpendicular to* ${V_{j}}$ *in* $V_{j+1}$, *and* $V_{j+1}=V_{j}\oplus W_{j}$. *Therefore*

$$2^{jd/2}\psi^{(j)}_{p}\bigl(2^{j}x-k \bigr), \quad k\in\mathbb {Z}, p=1,2,\ldots,2^{d}-1, $$

*is an orthonormal basis for* $H^{s}(\mathbb {R}^{d})$.

### Proof

First,we show that $\psi_{j,k,p}^{(j)}\bot V_{j}$, for all $k\in\mathbb {Z}^{d}$ and $p=1,2,\dots, 2^{d}-1$. Indeed,

$$\begin{aligned} & (2\pi)^{d}\bigl\langle \psi_{j,k,p}^{(j)}(x), \varphi_{j,k}^{(j)}(x)\bigr\rangle _{s} \\ &\quad=(2\pi)^{d}\bigl\langle 2^{jd/2}\psi_{p}^{(j)} \bigl(2^{j}x-k_{1}\bigr),2^{jd/2}\varphi _{j,k}^{(j)}\bigl(2^{j}x-k_{2}\bigr)\bigr\rangle _{s} \\ &\quad= \int_{\mathbb {R}^{d}}\bigl(1+ \Vert \xi \Vert ^{2} \bigr)^{s}\mathcal {F}\bigl(2^{jd/2}\psi _{p}^{(j)} \bigl(2^{j}\xi-k_{1}\bigr)\bigr)\overline{\mathcal {F} \bigl(2^{jd/2}\varphi _{j,k}^{(j)}\bigl(2^{j} \xi-k_{2}\bigr)\bigr)}\,d\xi \\ &\quad=2^{-jd} \int_{\mathbb {R}^{d}}\bigl(1+ \Vert \xi \Vert ^{2} \bigr)^{s}\hat{\psi}_{p}^{(j)}\bigl(2^{-j} \xi \bigr)\overline{\hat{\varphi}^{(j)}\bigl(2^{-j}\xi \bigr)}e^{2^{-j}\langle\xi ,(k_{2}-k_{1})\rangle}\,d\xi \\ &\quad= \int_{\mathbb {R}^{d}}\bigl(1+2^{2j} \Vert \xi \Vert ^{2}\bigr)^{s}m_{p}^{(j+1)}(\xi/2)\hat{\varphi}^{(j+1)}(\xi/2) \\ &\qquad{} \times\overline{m_{0}^{(j+1)}(\xi/2)\hat{\varphi}^{(j+1)}(\xi /2)}e^{2^{-j}\langle\xi,(k_{2}-k_{1})\rangle}\,d\xi \\ &\quad= \int_{\mathbb {T}^{d}}\sum_{l\in\mathbb {Z}^{d}} \bigl(1+2^{2j} \Vert \xi+2\pi l \Vert ^{2} \bigr)^{s}m_{p}^{(j+1)}(\xi/2+\pi l)\hat{\varphi}^{(j+1)}(\xi/2+\pi l) \\ & \qquad{}\times\overline{m_{0}^{(j+1)}(\xi/2+\pi l)\hat{\varphi}^{(j+1)}(\xi/2+\pi l)}e^{2^{-j}\langle\xi,(k_{2}-k_{1})\rangle}\,d\xi \\ &\quad = \int_{\mathbb {T}^{d}} \Biggl[\sum_{q=0}^{2^{d}-1}m_{p}^{(j+1)}( \xi/2+\gamma _{q}\pi)\overline{m_{0}^{(j+1)}(\xi/2+ \gamma_{q}\pi)} \Biggr]e^{2^{-j}\langle\xi,(k_{2}-k_{1})\rangle}\,d\xi \end{aligned}$$

by Proposition 2.1. This expression is equal to zero because matrix (6) is unitary. Similarly, we can show that $W_{j,p_{1}}\bot W_{j,p_{2}}$ for all $p_{1},p_{2}\in\{1,2,\dots,2^{d}-1\} $.

We know show that $V_{j+1}=V_{j}\oplus W_{j,1}\oplus W_{j,2}\oplus\cdots \oplus W_{j,2^{d}-1}$ for any $f\in V_{j+1}$. We write

$$\hat{f}(\xi)=B\bigl(2^{-j-1}\xi\bigr)\hat{\varphi}^{(j+1)} \bigl(2^{-j-1}\xi\bigr). $$

We will demonstrate that there exist $2\pi\mathbb {Z}^{d}$-periodic functions $G(2^{-j}\xi)$ and $H_{p}(2^{-j}\xi)$ such that

$$\hat{f}(\xi)=G\bigl(2^{-j}\xi\bigr)\hat{\varphi}^{(j)} \bigl(2^{-j}\xi\bigr)+\sum_{p=1}^{2^{d}-1}H_{p} \bigl(2^{-j}\xi\bigr)\hat{\psi}_{p}^{(j)} \bigl(2^{-j}\xi\bigr). $$

Now, we have

$$\begin{aligned} B(\xi/2)\hat{\varphi}^{(j+1)}(\xi/2)&=G(\xi)\hat{\varphi}^{(j)}( \xi)+\sum_{p=1}^{2^{d}-1}H_{p}(\xi) \hat{\psi}_{p}^{(j)}(\xi) \\ &=G(\xi)m_{0}^{(j+1)}(\xi/2)\hat{\varphi}^{(j+1)}(\xi/2)+ \sum_{p=1}^{2^{d}-1}H_{p}( \xi)m_{p}^{(j+1)}(\xi/2)\hat{\varphi}^{(j+1)}(\xi/2). \end{aligned}$$

It follows that

$$B(\xi/2)=G(\xi)m_{0}^{(j+1)}(\xi/2)+\sum _{p=1}^{2^{d}-1}H_{p}(\xi )m_{p}^{(j+1)}( \xi/2). $$

By the periodicity ($2\pi\mathbb {Z}^{d}$-periodic) of *G* and $H_{p}$ we have

$$B(\xi/2+\gamma_{q}\pi)=G(\xi)m_{0}^{(j+1)}(\xi/2+ \gamma_{q}\pi)+\sum_{p=1}^{2^{d}-1}H_{p}( \xi)m_{p}^{(j+1)}(\xi/2+\gamma_{q}\pi) $$

for $q=0,1,\dots,2^{d}-1$. This completes proof. □

## Multivariate box spline

Now we give an example of multivariate box splines in a Sobolev space. Using them, we construct a wavelet in $H^{s}(\mathbb {R}^{d})$.

Let *D* be the direction matrix of order $d\times\sum^{d+1}_{i=1}m_{i}, m_{i}\in\mathbb {N}_{0}, \forall i$, whose column vectors consist of $(m_{1},m_{2},\dots,m_{d+1})$ copies of the following $d+1$ column vectors: $(1,0,\dots,0)^{T}, (0,1,0,\dots,0)^{T},\dots,(0,0,\dots,1)^{T}$, and $(1,1,\dots,1)^{T}$.

Fix $s\geq0$ and the natural numbers $(m_{1},m_{2},\dots,m_{d+1})$ such that

$$\bigl\{ m[D]:=\min\{m_{i}+m_{j}: i\neq j \text{ for all } i,j=1,2,\dots ,d+1 \} \bigr\} +\frac{1}{2}>s. $$

Let $M_{m_{1},m_{2},\dots,m_{d+1}}$ be a multivariate box spline function defined in terms of the Fourier transform by

$$\widehat{M}_{m_{1},m_{2},\dots,m_{d+1}}(\xi)=\prod_{j=1}^{d+1} \biggl(\frac {1-e^{-i\langle k_{j},\xi\rangle}}{i\langle k_{j},\xi\rangle} \biggr)^{m_{j}},\quad k_{j}\in D, m_{j}\in\mathbb {N}_{0}, \forall j. $$

The multivariate box spline $M_{m_{1},m_{2},\dots,m_{d+1}}$ belongs to $C^{m[D]-1}$, where $m[D]+1$ is the minimum number of columns that can be discarded from *D* to obtain a matrix of $\operatorname{rank}< d$ (see [15]).

For

$$W_{m_{1},m_{2},\dots,m_{d+1}}^{(j)}(\xi):=\sum_{l\in\mathbb {Z}^{d}} \bigl(1+2^{2j} \Vert \xi+2\pi l \Vert ^{2} \bigr)^{s} \bigl\vert \widehat{M}_{m_{1},m_{2},\dots,m_{d+1}}(\xi+2\pi l) \bigr\vert ^{2}, $$

it is known that there exist $c,C\geq0$ such that

$$0\leq c\leq\sum_{l\in\mathbb {Z}^{d}} \bigl\vert \widehat{M}_{m_{1},m_{2},\dots ,m_{d+1}}(\xi+2\pi l) \bigr\vert ^{2}\leq C< \infty. $$

Considering $\xi:=(\xi_{1},\xi_{2},\dots,\xi_{d})$ and $l:=(l_{1},l_{2},\dots ,l_{d})$, we have

7 $$\begin{aligned} &\sum_{l\in\mathbb {Z}^{d}}\bigl(1+2^{2j} \Vert \xi+2\pi l \Vert ^{2}\bigr)^{s} \bigl\vert \widehat {M}_{m_{1},m_{2},\dots,m_{d+1}}(\xi+2\pi l) \bigr\vert ^{2} \\ &\quad =\sum_{(l_{1},l_{2},\dots,l_{d})\in\mathbb {Z}^{d}}\Biggl(1+2^{2j} \sum_{i=1}^{d} \vert \xi _{i}+2 \pi l_{i} \vert ^{2}\Biggr)^{s} \bigl\vert \widehat{M}_{m_{1},m_{2},\dots,m_{d+1}}(\xi+2\pi l) \bigr\vert ^{2}. \end{aligned}$$

By mathematical induction we know that, for positive real numbers $x_{i}$, $i=1,\dots,d$,

8 $$\begin{aligned} \Biggl(\sum_{`i=1}^{d}x_{i} \Biggr)^{m}\leq d^{m} \Biggl(\sum _{i=1}^{d}(x_{i})^{m} \Biggr),\quad x_{i}\in\mathbb {R}_{+}. \end{aligned}$$

From (7) and (8) we have

$$\begin{aligned} &\sum_{(l_{1},l_{2},\ldots,l_{d})\in\mathbb {Z}^{d}} \Biggl(1+2^{2j}\sum _{i=1}^{d} \vert \xi _{i}+2\pi l_{i} \vert ^{2} \Biggr)^{s} \bigl\vert \widehat{M}_{m_{1},m_{2},\ldots,m_{d+1}}(\xi+2\pi l) \bigr\vert ^{2} \\ &\quad\leq(d+1)^{s} \Biggl(c+2^{2js}\sum _{(l_{1},l_{2},\ldots,l_{d})\in\mathbb {Z}^{d}} \Biggl(\sum_{i=1}^{d} \vert \xi_{i}+2\pi l_{i} \vert ^{2s} \Biggr) \bigl\vert \widehat {M}_{m_{1},m_{2},\ldots,m_{d+1}}(\xi+2\pi l) \bigr\vert ^{2} \Biggr) \\ &\quad\leq(d+1)^{s} \Biggl(c+2^{2js}C'\sum _{(l_{1},l_{2},\ldots,l_{d})\in\mathbb {Z}^{d}} \Biggl(\sum_{i=1}^{d} \bigl\vert \widehat{M}_{m_{i}-s,m_{d+1}}(\xi+2\pi l) \bigr\vert ^{2} \Biggr) \Biggr) \\ &\quad\leq C_{j}< +\infty, \end{aligned}$$

where $C',C_{j}>0$, and $m_{i}-s,m_{d+1}$ is the *i*th term subtracted by *s*. Hence we have the following:

### Lemma 3.1

*There exists two constants* $c_{j}$ *and* $C_{j}$ *such that*

$$0< c_{j}\leq W_{m_{1},m_{2},\ldots,m_{d+1}}^{(j)}(\xi)\leq C_{j}< +\infty. $$

Now, for every $j\in\mathbb {Z}$, we define

9 $$\begin{aligned} \hat{\varphi}^{(j)}(\xi)=\frac{\widehat{M}_{m_{1},m_{2},\ldots,m_{d+1}}(\xi )}{\sqrt{W_{m_{1},m_{2},\ldots,m_{d+1}}^{(j)}(\xi)}}. \end{aligned}$$

Now we find a $2\pi\mathbb {Z}^{d}$-periodic function $m_{0}^{(j)}\in L^{2}(\mathbb {Z}^{d})$ for which the scaling relation (5) holds:

$$\varphi^{(j)}(2\xi)=m_{0}^{(j+1)}(\xi) \varphi^{(j+1)}(\xi). $$

From (9) we get

$$\begin{aligned} m_{0}^{(j+1)}(\xi)&=\frac{\widehat{M}_{m_{1},m_{2},\ldots,m_{d+1}}(2\xi )}{\widehat{M}_{m_{1},m_{2},\ldots,m_{d+1}}(\xi)}\sqrt{ \frac {W_{m_{1},m_{2},\ldots,m_{d+1}}^{(j+1)}(\xi)}{W_{m_{1},m_{2},\ldots ,m_{d+1}}^{(j)}(2\xi)}} \\ &=\prod_{i=1}^{d+1} \biggl( \frac{1+e^{-i\langle k_{i},\xi\rangle}}{2} \biggr)^{m_{i}}\sqrt{\frac{W_{m_{1},m_{2},\ldots,m_{d+1}}^{(j+1)}(\xi )}{W_{m_{1},m_{2},\ldots,m_{d+1}}^{(j)}(2\xi)}}. \end{aligned}$$

Finally, let us construct wavelets associated with the scaling function $\varphi^{(j)},j\in\mathbb {Z}$. We define the $2\pi\mathbb {Z}^{d}$-periodic functions $m_{p}^{(j)}$, $p=1,2,\ldots,2^{d-1}$, by

$$m_{p}^{(j)}(\xi)=e^{-i\langle\gamma_{p},\xi\rangle}\mathcal {L}_{p}^{(j)}(2\xi )\overline{m_{0}^{(j+1)}( \xi+\gamma_{p}\pi)}, $$

where the trigonometric polynomial $\mathcal {L}_{p}^{(j)}$ is to be chosen such that $m_{p}^{(j)}$ satisfies (6) for all *p*.

### Proposition 3.2

*Suppose* $\varphi^{(j)}$ *is a scaling function for an MRA* $V_{j}, j\in\mathbb {Z}$, *of* $H^{s}(\mathbb {R}^{d})$ *and* $m_{0}^{(j)}$ *is the associated low pass filter*. *Then the distributions* $2^{j/2}\psi ^{(j)}(2^{j}x-k), j\in\mathbb {Z}, k\in\mathbb {Z}^{d}$, *are an orthonormal basis for* $H^{s}(\mathbb {R}^{d})$ *if and only if*

$$\hat{\psi}_{p}^{(j)}(2\xi)=e^{-i\langle\gamma_{p},\xi\rangle}\mathcal {L}_{p}^{(j)}(2\xi)\overline{m_{0}^{(j+1)}( \xi+\gamma_{p}\pi)}\hat{\varphi}^{(j+1)}(\xi),\quad \forall p=1,2, \ldots,2^{d-1}, $$

*a*.*e*. *on* $\mathbb {R}^{d}$ *for some* $2\pi\mathbb {Z}^{d}$-*periodic function* $\mathcal {L}_{p}^{(j)}$ *such that*

$$\bigl\vert \mathcal {L}_{p}^{(j)}(\xi) \bigr\vert =1,\quad \forall p=1,2,\ldots,2^{d-1}, \textit{a.e. } \xi\in\mathbb {T}^{d}. $$

### Proof

From Proposition 2.1 we get

10 $$\begin{aligned} \sum_{k\in\mathbb {Z}^{d}} \bigl(1+2^{2j} \Vert \xi+2k\pi \Vert ^{2} \bigr)^{s} \bigl\vert \hat{\psi}_{p}^{(j)}(\xi+2k\pi) \bigr\vert ^{2}=1,\quad \forall p=1,2,\ldots,2^{d-1}. \end{aligned}$$

Now, we have only to verify the density condition

$$\lim_{j\rightarrow+\infty} \bigl\vert \varphi^{(j)} \bigl(2^{-j}\xi\bigr) \bigr\vert =\bigl(1+ \Vert \xi \Vert ^{2}\bigr)^{-s/2}. $$

By definition,

$$W_{m_{1},m_{2},\ldots,m_{d+1}}^{(j)}\bigl(2^{-j}\xi\bigr)=\sum _{k\in\mathbb {Z}^{d}}\bigl(1+ \bigl\Vert \xi+2^{j+1}\pi k \bigr\Vert ^{2}\bigr)^{s} \bigl\vert \widehat{M}_{m_{1},m_{2},\ldots,m_{d+1}} \bigl(2^{-j}\xi +2\pi k\bigr) \bigr\vert ^{2}. $$

The term associated with $k=0$ converges to $(1+\|\xi\|^{2})^{s}$. Using the estimates

$$\bigl\vert \widehat{M}_{m_{1},m_{2},\ldots,m_{d+1}}\bigl(2^{-j}\xi+2\pi k\bigr) \bigr\vert = \Biggl\vert \prod_{j'=1}^{d+1} \biggl(\frac{1-e^{-i\langle k_{j'},2^{-j}\xi\rangle }}{i\langle k_{j'},2^{-j}\xi+2\pi k\rangle} \biggr)^{m_{j'}} \Biggr\vert $$

for $\xi=(\xi_{1},\xi_{2},\ldots,\xi_{d})$ and

$$\begin{aligned} & \Biggl\vert \prod_{j'=1}^{d+1} \biggl( \frac{1-e^{-i\langle k_{j'},2^{-j}\xi \rangle}}{i\langle k_{j'},2^{-j}\xi+2\pi k\rangle} \biggr)^{m_{j'}} \Biggr\vert \\ & \quad\leq \Biggl(\prod_{j'=1}^{d+1} \biggl\vert \frac{\sin(2^{-(j+1)}\xi _{j'})}{2^{-j-1}\xi_{j'}+\pi k} \biggr\vert ^{m_{j'}} \Biggr) \biggl( \biggl\vert \frac {\sin(2^{-(j+1)}\sum^{d}_{j'=1}\xi_{j'})}{2^{-j-1}\sum^{d}_{j'=1}\xi _{j'}+d\pi k} \biggr\vert ^{m_{d+1}} \biggr) \\ &\quad \leq\frac{2^{-(j+1)(\sum^{d+1}_{j'=1}m_{j'})} (\prod_{j'=1}^{d} \vert \xi_{j'} \vert ^{m_{j'}} ) ( \vert \sum^{d}_{j'=1}\xi _{j'} \vert ^{m_{d+1}} )}{ \vert k \vert ^{\sum^{d+1}_{j'=1}m_{j'}}} \end{aligned}$$

for $2^{-(j+1)} (\prod_{j'=1}^{d}|\xi_{j'}|^{m_{j'}} ) ( \vert \sum^{d}_{j'=1}\xi_{j'} \vert ^{m_{d+1}} )<1$ and $k=0$, we see that, as $j\rightarrow+\infty$, the sum of the other terms converges to 0. The conclusion follows easily.

If $\psi_{p}^{(j)}$ is an orthonormal wavelet, then the orthonormality of $\{2^{j/2}\psi_{p}^{(j)}(2^{j}\cdot-k): j\in\mathbb {Z}, k\in\mathbb {Z}^{d}, p=1,2,\ldots,2^{d-1}\}$ gives us

$$\begin{aligned} 1={}&\sum_{k\in\mathbb {Z}^{d}} \bigl(1+2^{2j} \Vert \xi+2k\pi \Vert ^{2} \bigr)^{s} \bigl\vert \hat{\psi}_{p}^{(j)}(\xi+2k\pi) \bigr\vert ^{2} \\ ={}&\sum_{k\in\mathbb {Z}^{d}} \bigl(1+2^{2j} \Vert \xi+2k \pi \Vert ^{2} \bigr)^{s} \bigl\vert \mathcal {L}_{p}^{(j)}(\xi) \bigr\vert ^{2} \bigl\vert \hat{\varphi}^{(j+1)}(\xi/2+k\pi) \bigr\vert ^{2} \\ &{} \times \bigl\vert {m_{0}^{(j+1)}(\xi/2+k\pi+ \gamma_{p}\pi)} \bigr\vert ^{2} \\ ={}& \bigl\vert \mathcal {L}_{p}^{(j)}(\xi) \bigr\vert ^{2} \Biggl(\sum_{l\in\mathbb {Z}^{d}} \bigl(1+2^{2(j+1)} \Vert \xi/2 +2l\pi \Vert ^{2} \bigr)^{s} \bigl\vert \hat{\varphi}^{(j+1)}(\xi/2+2l\pi ) \bigr\vert ^{2} \\ &{} \times\sum_{q=1}^{2^{d}-1} \bigl\vert {m_{0}^{(j+1)}(\xi/2+\gamma_{q}\pi)} \bigr\vert ^{2}+\sum_{l\in\mathbb {Z}^{d}} \bigl(1+2^{2(j+1)} \Vert \xi/2 +2l\pi+\gamma_{q}\pi \Vert ^{2} \bigr)^{s} \\ & {}\times \bigl\vert \hat{\varphi}^{(j+1)}(\xi/2+2l\pi+ \gamma_{q}\pi ) \bigr\vert ^{2} \bigl\vert {m_{0}^{(j+1)}(\xi/2)} \bigr\vert ^{2} \Biggr) \\ ={} &\bigl\vert \mathcal {L}_{p}^{(j)}(\xi) \bigr\vert ^{2} \Biggl(\sum_{q=0}^{2^{d}-1} \bigl\vert {m_{0}^{(j+1)}(\xi /2+\gamma_{q}\pi)} \bigr\vert ^{2} \Biggr)= \bigl\vert \mathcal {L}_{p}^{(j)}( \xi) \bigr\vert ^{2},\quad p=1,2,\ldots,2^{d-1}, \end{aligned}$$

for a.e. $\xi\in\mathbb {T}^{d}$ and $\gamma_{q},\gamma_{p}\in E_{d}$, which finishes our proof. □

## Conclusion

In this paper, we have successfully generalized MRA over higher-dimensional Sobolev spaces by giving orthonormality and density conditions. Further, we constructed nonseparable orthonormal wavelets in a higher-dimensional Sobolev space by using multivariate box splines. The main obstacle in constructing wavelets is constructing low-pass and high-pass filters with the help of multivariate box splines, which satisfy the condition of orthonormality in $H^{s}(\mathbb {R}^{d})$ for every scale *j* (because the $H^{s}$-norm is not dilation invariant).
